# Usefulness of 2‐D shear wave elastography for the diagnosis of inguinal lymph node metastasis of malignant melanoma and squamous cell carcinoma

**DOI:** 10.1111/1346-8138.15545

**Published:** 2020-08-13

**Authors:** Yu Kawahara, Yaei Togawa, Yosuke Yamamoto, Seiichiro Wakabayashi, Hiroyuki Matsue, Kazuhiro Inafuku

**Affiliations:** ^1^ Department of Dermatology Kimitsu Chuo Hospital Kisarazu Japan; ^2^ Department of Dermatology Chiba University Graduate School of Medicine Chiba Japan

**Keywords:** 2‐D shear wave elastography, lymph node, malignant melanoma, region of interest, squamous cell carcinoma

## Abstract

We used 2‐D shear wave elastography to quantify lymph node hardness, from the shear wave velocity, to determine the presence or absence of metastatic lymphadenopathy in the inguinal lymph nodes of five patients with malignant melanoma and squamous cell carcinoma. The shear wave velocity accurately identified all cases of metastasis confirmed by histology, compared with two false‐positive and one false‐negative finding with positron emission tomography/computed tomography. 2‐D shear wave elastography would be useful to evaluate inguinal lymph node metastasis.

## Introduction

Contrast‐enhanced computed tomography (CT) and positron emission tomography (PET)/CT are generally used to evaluate lymph node metastasis from skin cancer. More recently, strain (SE) and shear wave elastography (SWE) have been developed to provide an objective measure of lymph node hardness.^1^, ^2^, ^3^ However, signal acquisition and interpretation with SE are influenced by the examiner’s experience and results can differ considerably between examiners.^2^ On the other hand, SWE uses an acoustic radiation force (ARF) as the excitation impulse, with the propagation velocity of the transverse elastic wave (shear wave) generated by irradiation of convergent ultrasonic pulses that deform the target tissue.^2^ As such, tissue hardness can be quantified by the velocity of the shear wave velocity (SWV) within a region of interest (ROI) and can be measured by repeated application of a B‐mode ultrasound pulse.^1^, ^3^, ^4^ Of note, SWV measurement is not affected by an examiner’s experience, conducted by placing the probe on the body surface for a few seconds.^4^, ^5^ Within normal tissues, the velocity of the ultrasound wave can be transmitted at a velocity of up to 1500 m/s, with the resulting shear wave being transmitted at a relatively slow velocity of 1–10 m/s. As the shear wave propagates faster in harder tissues, the SWV provides an objective measure of tissue hardness.^6^

**Figure 1:**
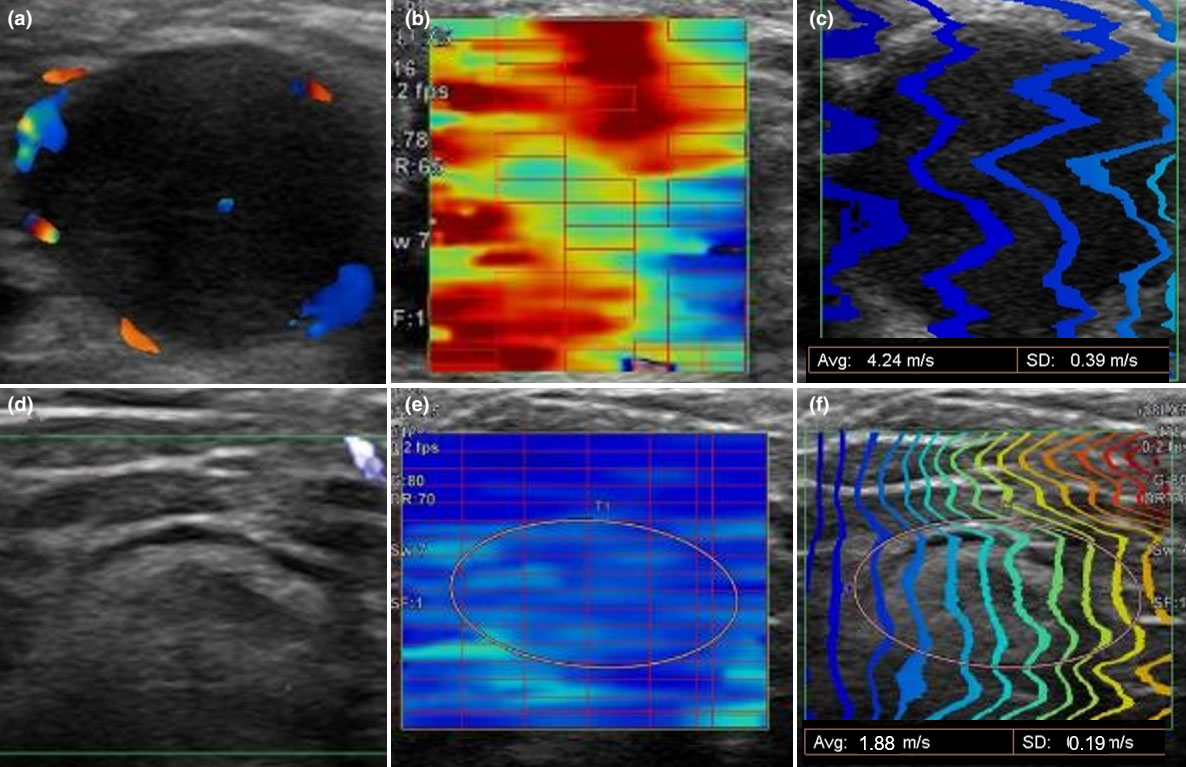
Principle of 2‐D shear wave elastography (2DSWE) (for case 1 and 2), where bluish tones correspond to deformable tissues (soft). (a) Color Doppler and (b,c) 2DSWE images of the metastatic lymph node for case 1, and (d) superb microvascular imaging (SMI) and (e,f) 2DSWE images of the non‐metastatic lymph node for case 2. The Aplio i800 displays the speed of the shear wave in two types of images: (b,e) color display and (c,f) arrival time contour display. In the initial setting of the color display, non‐deformable tissue (hard), in which the shear wave velocity is high, is shown in red, while deformable tissue (soft), in which the shear wave velocity is low, is shown in blue. (a) Color Doppler image of a metastatic lymph node with disrupted capsule and extranodal invasion. Circular low echoic lesion, 14.3 × 11.9 mm in size, with increase in blood flow signal at the margin. (b) This lesion showed red on the color display mode, (c) with wide interval waves in the arrival time counter display mode, indicative of a high velocity. (d) In contrast, in the SMI image of the lymph node that had no histopathological metastasis, an oval lesion, 11.7 × 4.8 mm in size, showed low echoic margins with a slightly higher echoic internal areas without an increase in the blood flow signal at the margin. (e) This is shown in blue, (f) with narrow interval waves indicative of a slow velocity. SD, standard deviation.

Quantitative SWE modalities include conventional point SWE (pSWE) and the new 2‐D SWE (2DSWE).^5^, ^6^, ^7^, ^8^, ^9^ 2DSWE uses ARF beams to generate shear waves in a full field of view, with the SWV measured from sequential points of measurement, while pSWE excites the target tissue in a single focal location.^5^, ^7^, ^8^ 2DSWE incorporates conventional B‐mode ultrasound, with a color‐coded elasticity map generated in real time (Fig. 1), to precisely locate the ROI for high‐quality measurements.^5^, ^8^

Although pSWE has been used to evaluate lymph node metastasis of head and neck tumors,^4^, ^10^, ^11^, ^12^, ^13^ little is known about 2DSWE. Therefore, our aim in this study was to evaluate the usefulness of 2DSWE to identify metastatic lymphadenopathy of skin cancer in the inguinal region.

## Methods

### Participants

Our case series included five patients (mean age, 75.6 years; range, 65–82; male : female ratio, 3:2) who underwent inguinal lymphadenectomy for lower limb malignant skin tumors, between October 2017 and August 2018, three with melanomas and two with squamous carcinomas (Table 1). The SWV of inguinal lymphadenopathy, measured in eight lymph nodes contributed by three patients with lower limb cellulitis, were used as negative controls.

**Table 1:** Background of the five cases and evaluation of lymphadenopathy on PET/CT and 2DSWE

Case	Case 1		Case 2		Case 3		Case 4	Case 5	
Age (years)/sex	81/F		70/M		65/M		80/M	82/F	
Cancer type	MM		MM		SCC		SCC	MM	
Primary lesion	Right buttock		Right sole		Right thigh		Right sole	Right hallux	
Stage	pT4aN2bM0 Stage III C		pT2bN0M0 Stage II A		pT2N0M0 Stage II		pT3N0M0 Stage III	pT4bN2aM0 Stage III C	
Inguinal surgery history	+		−		+		−	+	
Lymph node number	1	2	3	4	5	6	7	8	9
PET/CT	+	+	−	−	+	+	+	−	−
SUVmax	5.4	2.1	LoD	LoD	2.8	4.9	4.19	LoD	LoD
Sonographic features									
Long axis (mm)	14.3	7.7	35.6	11.7	19.0	21.6	40.7	14.9	6.6
Short axis (mm)	11.9	4.9	6.2	4.8	7.2	11.2	12.5	2.5	2.8
L/S ratio	1.2	1.6	5.7	2.4	2.6	1.9	3.3	6.0	2.4
Hilum	−	−	+	+	+	+	+	+	+
Peripheral flow	+	+	−	−	−	+	−	−	−
SWV (m/s)	4.24	5.14	2.34	1.88	1.67	1.45	2.30	2.04	4.81
Histological metastasis	+	+	−	−	−	−	−	−	+

2DSWE, 2‐D shear wave elastography; L/S ratio, long‐to‐short axis ratio; LoD, below the limit of detection; MM, malignant melanoma; PET/CT, positron emission tomography/computed tomography; SCC, squamous cell carcinoma; SUVmax, maximum standardized uptake value; SWV, shear wave velocity.

### Statement of ethics

This small clinical trial was approved by the Kimitsu Central Hospital ethics review board (approval no. 473). All patients provided informed consent to participate in our study.

### Assessment

Positron emission tomography/CT and 2DSWE of the regional lymph nodes in the groin area were performed preoperatively and compared with postoperative histological results. All 2DSWE assessments were performed by the same examiner, using the Aplio i800 ultrasound device (Canon Medical Systems, Otawara, Japan), with a 12–18‐MHz linear probe and a convex 5‐MHz probe. We performed 2DSWE along the cross‐sectional plane at the point of maximum diameter of the lymph node and SWV measurements were repeated several times until the difference between the two measurements was within 10%.

The ROI was set to the entire target area of suspected lymph node metastasis.

### Analysis

Nine lymph nodes that were palpable and sized more than 1 cm or had suspected metastasis detected on PET/CT or 2DSWE were eligible for analysis, with a histopathological image available for each.

## Results

Shear wave velocity measurements of the nine inguinal lymph nodes in the cancer group are shown in Table 1. Characteristics of B‐mode and Doppler ultrasound images, such as the long‐to‐short axis ratio and peripheral flow, were suggestive of metastases in lymph nodes 1 and 2 in case 1, and lymph node 6 in case 3. The metastasis status of lymph nodes was confirmed by histopathological assessment in two cases (cases 1 and 5) but not in the other three.

In case 1, both lymph node number 1 (SWV, 4.24 m/s) and 2 (SWV, 5.14 m/s) showed diffuse histopathological infiltration of tumor cells, with a ruptured capsule and extranodal infiltration. Case 5, lymph node number 9 (SWV, 4.81 m/s), showed micrometastatic invasion, occupying less than 1/30 of the lymph node, with no capsule disruption but with fibrosis of the stroma and hilum of this lymph node. The maximum SWV value in lymph nodes with no histological evidence of metastasis was 2.34 m/s, with a minimum SWV of 4.24 m/s in lymph nodes with evidence of metastasis. The SWV in the negative control group of eight lymph nodes with lower limb cellulitis ranged 1.43–2.32 m/s.

**Figure 2:**
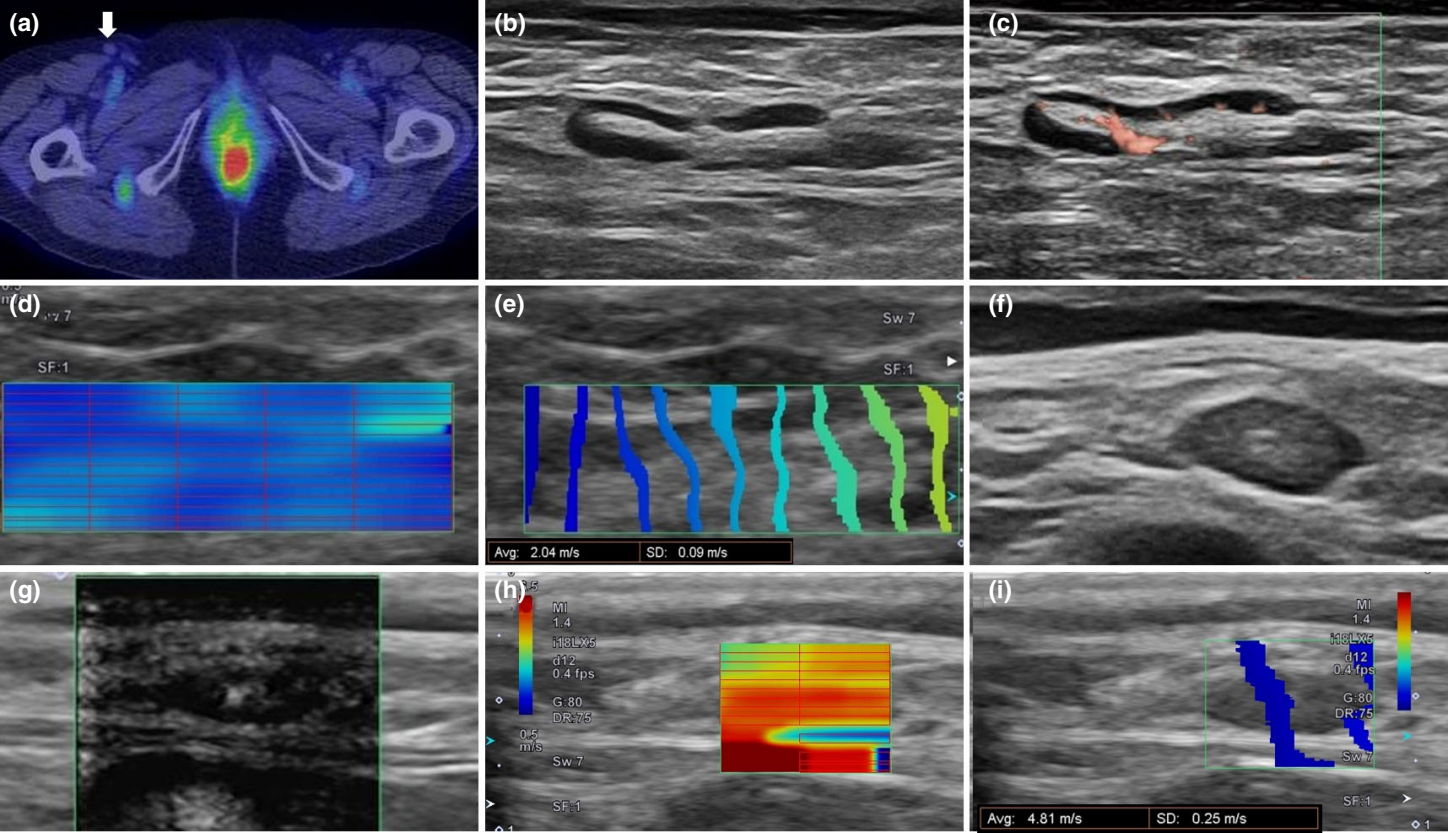
Comparison of positron emission tomography/computed tomography (PET/CT) and 2‐D shear wave elastography (2DSWE) images for case 5. (a) One large inguinal lymph node, with no histopathological evidence of metastasis (arrow), is identified on PET/CT with no significant ^18^F‐fluorodeoxyglucose accumulation. (b–e) Surface echo images of the same lymph node identified by PET/CT. (f–i) Surface echo images of the nearby lymph node with micrometastasis (occupying <1/30 of lymph node) not identified by PET/CT. (b) The echo image of the lymph node, shown with a clear cortical medullary border (14.9 mm × 2.5 mm), and (c) superb microvascular imaging (SMI), showing a blood flow increase signal in the hilum of lymph node. (d,e) 2DSWE images of the lymph node shown in (b). (d) The lymph node is shown in diffuse blue, indicating soft tissue. (e) Narrow intervals between the waves indicate that the waves are traveling slowly (SWV of 2.04 m/s, with negative histological metastasis). (f) Echo image of the nearby lymph node is shown with a clear cortical medullary border (6.6 mm × 2.8 mm) and (g) SMI, with no noticeable increase in the blood flow signal. (h,i) 2DSWE image of the lymph node shown in (f). (h) The lymph nodes are shown as red areas, indicating hard tissue. (i) The wide interval between the waves indicates that the waves are traveling quickly. SWV of 4.81 m/s (histological metastasis was positive). SD, standard deviation.

Among the nine lymph nodes in the cancer group, PET/CT yielded false‐positive findings in three lymph nodes (cases 3 and 4) and a false‐negative finding, with ^18^F‐fluorodeoxyglucose uptake below the limit of detection of standardized uptake value, in one lymph node (case 5; Fig. 2). By comparison, SWE did not yield either false‐negative or false‐positive results.

## Discussion

According to the PubMed search we conducted, from the date of inception to 11 April 2020, no reports assessing lymph node metastasis using 2DSWE were identified. All 104 reports of 2DSWE evaluated parenchymal organs (69 liver, 10 mammary glands, six thyroid and 19 others). Only one report documented the use of pSWE for the evaluation of inguinal lymph node metastasis of a breast tumor,^9^ with a cut‐off SWV value of more than 2.5 m/s having a sensitivity and specificity of 95% and 87%, respectively, for identifying metastasis. Furthermore, according to a systematic review and meta‐analysis that evaluated the diagnostic performance of pSWE for malignant cervical lymph nodes in cases of squamous cell carcinoma of the head and neck,^10^ four studies indicated that a high SWV (≥1.9–3.34 m/s) as a cut‐off value for lymph nodes is indicative of metastatic lymphadenopathy, with a sensitivity of 81% and specificity of 85%.^3^, ^11^, ^12^, ^13^

In our case series, which included three false‐positive and one false‐negative lymph node on PET/CT, the cut‐off SWV on 2DSWE for reactive lymphadenopathy without metastasis was 2.34 m/s or less, compared with 4.24 m/s or more for metastatic lymphadenopathy (Table 1). Considering the range of reported cut‐off SWV values of pSWE for the cervical (≥1.9–3.34 m/s)^4^, ^11^, ^12^, ^13^ and inguinal (>2.5 m/s) regions,^9^ the cut‐off value indicative of possible metastasis would fall between 2.34 and 4.24 m/s, although this would require further verification owing to our small sample size. Our findings, however, do indicate that 2DSWE may yield a higher specificity than PET/CT for early stage metastatic lymphadenopathy detection (Fig. 2).

Regarding ROI settings for 2DSWE, a study measured liver stiffness within a flexible ROI, which was larger than that for pSWE.^14^ For assessment of hardness of the cervical or inguinal lymph nodes, a small and fixed ROI has been used for pSWE, ranging 1–25 mm^2^ in size, with measures repeated 2–6 times.^3^, ^9^, ^11^, ^12^, ^13^ The ultrasound device used in our study was designed to allow examiners to optimize the ROI setting, generally encompassing the whole lymph node to be examined, with an area with parallel contour lines in a full field of view.^5^ Of note, when performing 2DSWE, it is necessary to confirm the presence or absence of calcification, as seen in tuberculosis and cat scratch disease, using B‐mode imaging, as well as to ensure that calcification is excluded from the ROI.

Small‐sized lymph node metastases associated with capsule destruction have been reported to be detectable by SWE.^14^ However, in our case, a high SWV value was observed at the stage of micrometastasis without capsule destruction but with stroma and hilar fibrosis of the lymph node (case 5; Fig. 2), presumably supporting the “priming the metastatic soil” hypothesis described in detail by Cox *et al*.^15^ that modification of the microenvironment including fibrosis can be initiated by a primary tumor before micrometastasis occurs.

Based on our assessment of nine lymph nodes in five patients, measured by 2DSWE, the hardness of the involved lymph nodes accurately identified the presence or absence of metastasis. PET/CT assessment yielded two false‐positive findings and one false‐negative, suggesting that SWV measurements using 2DSWE in lymph nodes would complement PET/CT. As a limitation, we have only evaluated false‐positive cases for squamous cell carcinoma and only five cases including melanoma have been studied, so more cases are needed to generalize our findings.

## Conflict of Interest

None declared.
